# 2505. Using an Electronic Medical Record Alert to Screen Inpatient Encounters for People with Hepatitis C

**DOI:** 10.1093/ofid/ofad500.2123

**Published:** 2023-11-27

**Authors:** Patrick Pryal, Walid El-Nahal, Thomas Grader-Beck, Grant Wilson, Brad Beatson, Oluwaseun Falade-Nwulia, Kelly Gebo, Stephen Berry

**Affiliations:** Johns Hopkins University School of Medicine, Baltimore, Maryland; Johns Hopkins School of Medicine, Baltimore, Maryland; Johns Hopkins University School of Medicine, Baltimore, Maryland; Johns Hopkins University School of Medicine, Baltimore, Maryland; Johns Hopkins University School of Medicine, Baltimore, Maryland; Johns Hopkins University, Baltimore, MD; Johns Hopkins, Baltimore, MD; Johns Hopkins University School of Medicine, Baltimore, Maryland

## Abstract

**Background:**

Despite the availability of effective therapy, rates of linkage and uptake of hepatitis C virus (HCV) treatment remain suboptimal. People with HCV are hospitalized at a rate 3.7 times that of the general population, presenting an opportunity for linkage to care during inpatient admissions. We designed two novel electronic medical record (EMR) alerts to identify patients with HCV in two hospitals, and report on the alerts’ test-performance characteristics.

**Methods:**

We developed two distinct EMR alerts and ran them silently on all adult hospitalizations at two academic medical centers in Baltimore, MD. Based on laboratory data, and diagnosis codes (Figure 1); the first alert identified all people ever infected with HCV (ever-alert); and, the second, only people with active HCV viremia (active-alert). We evaluated each alert’s positive predictive value (PPV) by manually reviewing all charts alerted during the study period. We evaluated alert sensitivity by reviewing a sample of consecutive hospital encounters during the same period to determine if they were alerted.

**Figure:**
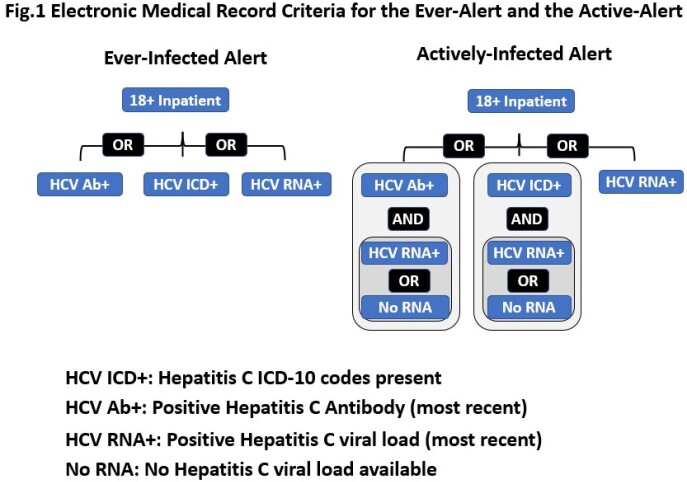

**Results:**

From 6/29/2022-8/9/2022, the alerts examined 7,519 adult hospital encounters. The ever-alert was triggered for 569 encounters; 563 of 569 were truly ever-infected (PPV 98.9% [95% CI 98.1%, 99.8%]), and 202 of 569 were actively-infected (PPV 35.5% [31.6%, 39.4%]), corresponding to an active infection prevalence of 202/7,519 (2.7%). The active-alert was triggered for 267 encounters; 188 of 267 were actively-infected (PPV 70.4% [65.0%-75.8%]), corresponding to a 2.5% prevalence of active infection. The consecutive chart review examined 1157 patients, 122 (10.5%) of whom were ever-infected, including 44 (3.8%) actively-infected. The ever-alert identified 106/122 (sensitivity 86.9% [80.9%, 92.9%]) and the active-alert identified 37/44 (sensitivity 84.1% [73.3%, 94.9%]) of these respectively (Table 1).

**Figure:**
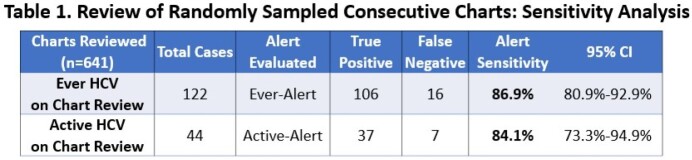

**Conclusion:**

An alert designed to identify only active infection has a comparable sensitivity (84.1% vs 86.9%) but significantly higher PPV (70.4% vs. 35.5%) for active infection than an alert designed to capture any infection (active or ever). These results support the use of an EMR alert for an HCV outreach program to link patients to care.

**Disclosures:**

**Oluwaseun Falade-Nwulia, MBBS ,MPH**, Abbvie Inc: Grant/Research Support|Gilead Sciences: Advisor/Consultant **Kelly Gebo, MD, MPH**, Pfizer: Advisor/Consultant|Spark HealthCare: Advisor/Consultant

